# Annexin A1 in blood mononuclear cells from patients with coronary artery disease: Its association with inflammatory status and glucocorticoid sensitivity

**DOI:** 10.1371/journal.pone.0174177

**Published:** 2017-03-22

**Authors:** Ida Bergström, Anna K. Lundberg, Simon Jönsson, Eva Särndahl, Jan Ernerudh, Lena Jonasson

**Affiliations:** 1 Department of Medical and Health Sciences, Division of Cardiovascular Medicine, Faculty of Health Sciences, Linköping University, Linköping, Sweden; 2 Department of Clinical Immunology and Transfusion Medicine, Department of Clinical and Experimental Medicine, Linköping University, Linköping, Sweden; 3 Department of Clinical Medicine, School of Health and Medical Sciences, and iRiSC - Inflammatory Response and Infection Susceptibility Centre, Faculty of Medicine and Health, Örebro University, Örebro, Sweden; Queen Mary University of London, UNITED KINGDOM

## Abstract

Annexin A1 (AnxA1) is a key player in resolution of inflammation and a mediator of glucocorticoid actions. In atherosclerotic tissue, increased expression of AnxA1 has been associated with protective plaque-stabilizing effects. Here, we investigated the expression of AnxA1 in peripheral blood mononuclear cells (PBMCs) from patients with coronary artery disease (CAD). Blood was collected from 57 patients with stable CAD (SCAD) and 41 healthy controls. We also included a minor group (n = 10) with acute coronary syndrome (ACS). AnxA1 mRNA was measured in PBMCs. Expression of AnxA1 protein (total and surface-bound) and glucocorticoid receptors (GR) were detected in PBMC subsets by flow cytometry. Also, salivary cortisol, interleukin(IL)-6 and IL-10 in plasma, and LPS-induced cytokine secretion from PBMCs, with or without dexamethasone, were assessed. AnxA1 mRNA was found to be slightly increased in PBMCs from SCAD patients compared with controls. However, protein expression of AnxA1 or GRs in PBMC subsets did not differ between SCAD patients and controls, despite SCAD patients showing a more proinflammatory cytokine profile ex vivo. Only surface expression of AnxA1 on monocytes correlated with dexamethasone-mediated suppression of cytokines. In ACS patients, a marked activation of AnxA1 was seen involving both gene expression and translocation of protein to cell surface probably reflecting a rapid glucocorticoid action modulating the acute inflammatory response in ACS. To conclude, surface expression of AnxA1 on monocytes may reflect the degree of glucocorticoid sensitivity. Speculatively, “normal” surface expression of AnxA1 indicates that anti-inflammatory capacity is impaired in SCAD patients.

## Introduction

Annexin A1 (AnxA1), formerly called lipocortin-1, is a key player in the resolution of inflammation and known as a mediator of the anti-inflammatory effects of glucocorticoids [1]. It is abundantly expressed in innate immune cells under normal conditions [2, 3]. In experimental models, AnxA1 or AnxA1-derived peptides exert a broad range of anti-inflammatory effects in monocytes, involving transcriptional changes as well as rapid post-translational effects [4, 5]. A number of studies have also shown that glucocorticoids induce de novo synthesis as well as translocation of AnxA1 to the cell surface in peripheral blood mononuclear cells (PBMCs) or isolated monocytes/macrophages [6–11]. The expression of AnxA1 in circulating PBMCs may thus serve as an indicator of anti-inflammatory actions and glucocorticoid sensitivity.

An imbalance between pro- and anti-inflammatory actions is believed to be important in the development and control of atherosclerosis [12, 13]. Several components of the immune system are involved in the inflammatory process of the arterial wall, including early recruitment and activation of monocytes to the intima followed by induction of the adaptive immune response [14, 15]. The pro-inflammatory milieu in atherosclerotic lesions is also mirrored in the circulation of patients with atherosclerotic disease. Several studies have described raised plasma levels of inflammatory cytokines and enhanced activation of PBMCs in patients with coronary artery disease (CAD), particularly in those with unstable conditions of the disease [12]. The presence of anti-inflammatory mediators, on the other hand, is less well documented. Only a few studies have investigated the expression of AnxA1 in atherosclerotic tissue [16–18]. A proteomics analysis of human coronary arteries demonstrated increased levels of AnxA1 in atherosclerotic tissue compared with non-atherosclerotic tissue and further validation by immunohistochemistry suggested that AnxA1 was expressed by macrophages in the intima [16]. Interestingly, two studies of human carotid plaques reported that AnxA1 expression was increased in plaques from asymptomatic patients compared with plaques from symptomatic patients, thus indicating an association between AnxA1 and plaque stabilization [17, 18]. Recently, in vivo administration of AnxA1 fragment Ac2-26 was shown to stabilize advanced atherosclerotic lesions in a mouse model [19].

In the present study, we compared the expression of AnxA1 in PBMCs from CAD patients and healthy subjects and related it to inflammatory status. Since previous studies have raised the question about insufficient glucocorticoid actions in CAD patients [20, 21], we also investigated whether AnxA1 expression in PBMCs correlated with cortisol levels, glucocorticoid receptor (GR) expression and glucocorticoid sensitivity.

## Material and methods

### Study populations

The study population consisted of 57 patients with stable CAD (SCAD) and 41 healthy controls. We also included a small group of 10 patients with acute coronary syndrome (ACS) from whom blood samples were collected within 24 h from admission, always prior to coronary angiography. In SCAD patients, blood samples were collected 6–12 months after an ACS event. All patients were consecutively recruited at the Department of Cardiology, University Hospital, Linköping, Sweden. ACS was defined as non-ST elevation myocardial infarction based on typical ECG changes (ST-T segment depression and/or T-wave inversion) and elevated troponins. In post-ACS patients, Patients were not included if they were over 75 years of age, had severe heart failure, immunologic disorders, neoplastic disease, evidence of acute or recent (< 2 months) infection, recent major trauma, surgery or revascularization procedure, and treatment with immunosuppressive or anti-inflammatory agents (except low-dose aspirin). As a healthy control group, age and sex matched subjects from the same area (region of Östergötland, Sweden) were randomly invited from the Swedish Population Register to participate in the study. The control subjects were anamnestically healthy without fulfilling any exclusion criteria, however use of statins or antihypertensive drugs for primary prevention was allowed. The study was conducted in accordance with the ethical guidelines of the Declaration of Helsinki, and the research protocol was approved by the Ethical Review Board of Linköping. Written informed consent was obtained from all study participants.

### Numbers and proportions of circulating leukocyte subsets

Numbers of leukocytes, monocytes, CD4^+^ T cells, CD8^+^ T cells, and NK cells were analyzed by flow cytometry in EDTA whole blood. As previously described [22], the number of cells/μL was determined by using Trucount^™^ tubes (BD Biosciences, San José, CA, USA) containing an exact number of lyophilized beads and a combination of monoclonal antibodies (CD3-FITC (clone SK7), CD4-PE-Cy7 (clone SK3), CD8-APC-H7 (clone SK1), CD45-PerCP (clone 2D1), CD56-PE (clone NCAM16.2), all from BD Biosciences. Briefly, antibodies were added to whole blood and samples were incubated for 15 min at room temperature (RT) in the dark. Erythrocytes were lysed with FACS Lysing Solution (BD Biosciences) for 15 min at RT in the dark, and samples were then immediately analyzed on a FACSCanto II (BD Biosciences) equipped with 3 lasers; a blue 488 nm, a red 633 nm and a violet 405 nm. Acquisition of samples was stopped when 30 000 cells were collected in the lymphocyte gate or, if few events, after 240 sec. Data were analyzed and subpopulations gated with FACSDiva 6.1.3 software (BD Biosciences).

### Flow cytometric determination of AnxA1 and GR expression in circulating monocytes and lymphocyte populations

For the assessment of AnxA1 protein expression in leukocyte subsets, EDTA whole blood (100 μL) was incubated with CD3-APC/H7 (clone SK7), CD4-V450 (clone RPA-T4), CD8-AF 700 (clone RPA-T8) (all from BD Biosciences), CD14-PeCy7 (clone M5E2; Biolegend, Nordic Biosite, Täby, Sweden), and CD56-APC (clone B159; BD Biosciences) for 15 min at RT in the dark. Erythrocytes were lysed with FACS Lysing Solution (BD Biosciences) for 10 min at RT in the dark, after which the protocols for surface and total (i.e. surface and cytosolic) AnxA1 protein expression were separated.

To detect surface expression of AnxA1, cells were first washed with phosphate-buffered saline (PBS) + 0.5% Fetal Bovine Serum (FBS; PAA, Invitrogen, Carlsbad, CA, USA). After centrifugation, unspecific binding was blocked with goat serum (GIBCO, Invitrogen) for 10 min at RT in the dark, before stained with rabbit anti-AnxA1 (Invitrogen) for 15 min at RT in the dark. Cells were washed and then stained with the secondary goat anti-rabbit F(ab’)_2_-PE (Invitrogen) for 15 min at RT in the dark, washed and then permeabilized/fixed with Permeabilizing solution 2 (Perm 2, BD Biosciences) for 10 min at RT in the dark. After centrifugation cells were resuspended in 1% paraformaldehyde (PFA)/PBS and kept at 4°C in the dark until flow cytometry analysis.

For detection of total expression of AnxA1 protein, cells were permeabilized/fixed with Perm 2 for 10 min at RT in the dark. After centrifugation, unspecific binding was blocked with goat serum for 10 min at RT in the dark, where after cells were stained with rabbit anti-AnxA1 for 30 min at RT in the dark. After washing, cells were incubated with secondary antibody (goat anti-rabbit F(ab’)_2_-PE) for 30 min at RT in the dark, washed again and subsequently resuspended in 1% PFA/PBS and kept at 4°C in the dark until flow cytometry analysis. Samples without primary anti-AnxA1 were used as a control for background staining.

For intra-cellular assessment of GR-alpha and GR-beta expression in leukocyte subsets, EDTA whole blood (100 μL) was stained with CD3-APC/H7 (clone SK7), CD4-V450 (clone RPA-T4), CD8-AF 700 (clone RPA-T8), CD14-PeCy7 (clone M5E2), CD56-APC (clone B159) and incubated for 15 min at RT in the dark, thereafter erythrocytes were lysed with FACS Lysing Solution for 10 min at RT in the dark. Cells were permeabilized/fixed with Perm 2 for 30 min at 4°C and washed with Permeabilization buffer (Ebioscience, San Diego, CA, USA). Unspecific binding was blocked with goat serum for 10 min at RT in the dark, and thereafter cells were stained with rabbit anti-human GR-alpha or GR-beta antibodies (ThermoFischer, Waltham, MA, USA; PA1-516 and PA3-514 respectively) for 30 min in 4°C in the dark. After washing in Permeabilization buffer, cells were incubated with the secondary goat anti-rabbit F(ab’)_2_-PE for 30 min at RT, washed again in Permeabilization buffer, and finally resuspended in PBS with 0.5% FCS and kept at 4°C in the dark until flow cytometry analysis. If samples were not immediately analyzed, cells were instead resuspended in 1% PFA/PBS. Samples without primary anti-GR-α or anti-GR-β were used as a control for background staining.

All samples were analyzed using Beckman Coulter Gallios (Beckman Coulter, Miami Lakes, Florida, US). Analysis of samples was stopped when 5000 cells were collected in the monocyte gate. Data were analyzed and subpopulations gated using the Kaluza 1.2 software (Beckman Coulter).

### PBMC isolation and cell culture

PBMCs were isolated by density centrifugation (Ficoll-Paque) 400g x g for 40 min at RT, washed twice in PBS+ 0.5% FBS, and resuspended in RPMI 1640 medium supplemented with L-glutamine (Life Technologies, Carlsbad, California, USA), 10% FBS, 100 U/mL penicillin and 100 μg/mL streptomycin (Life Technologies) to a concentration of 10^6^ cells/mL. Cells were incubated with or without 100 ng/mL lipopolysaccharide (LPS) from *E*.*coli* (Sigma-Aldrich, St Louis, MO, USA,) alone or in combination with 10^−7^ M or 10^−8^ M dexamethasone (Sigma-Aldrich), for 19 h at 37°C in a humidified atmosphere with 5% of CO_2_. Cell supernatants and cells (snap frozen in liquid nitrogen) were collected, and stored at -80°C until analysis.

### AnxA1 mRNA in PBMCs

Total RNA was isolated both from freshly isolated PBMCs and from cultured PBMCs with MagMAX^™^-96 Total RNA Isolation Kit (Life Technologies), according to manufacturer's instructions. RNA (66 ng) was reversely transcribed by using high capacity cDNA reverse transcription kit with an RNAse inhibitor (Life Technologies) according to manufacturer's instructions. cDNA (1 μL) was amplified by RT-PCR reactions with 1× TaqMan Fast Universal PCR Mastermix (Life Technologies) in 96-well plates on an ABI 7500 Sequence Detector with SDS 1.3.1 software. TaqMan Gene Expression Assay kits (Life Technologies) for AnxA1, Hs00167549_m1 was used. Eukaryotic 18S rRNA (Part number: 4352930E) with an amplicon length of 187 bp served as endogenous control. The amount of expressed AnxA1 was calculated relative to the amount of rRNA in each sample and the ΔΔCT method were used according to the user bulletin no 2. Each sample was run in duplicates and a maximum deviation of 4% was allowed.

### LPS-induced ex vivo secretion of IL-1beta, IL-6, IL-10 and TNF in PBMC cell supernatants by luminex

Cell supernatants from cultured PBMCs, in the presence or absence of LPS, were assessed for IL-1beta, IL-6, IL-10, and TNF concentrations by multiplexed bead assay analysis using Human premixed multi-analyte kit (R&D Systems, Abingdon, UK). Manufacturer’s protocol was followed. Standard curve range was from 2.7–1987 pg/mL for IL-1beta, 3.0–2223 pg/mL for IL-6, 6.1–4474 pg/mL for IL-10, and 6.13–4470 pg/mL for TNF, respectively. Inter-assay coefficients of variation (CV %) for the analyses were 12.5, 18.3, 9.9, and 13.8 for IL-1beta, IL-6, IL-10, and TNF, respectively.

### Cytokines in plasma

IL-6 and IL-10 were analyzed according to the manufacturer´s instructions in EDTA-plasma using Human IL-6 QuantiGlo^®^ ELISA chemiluminesence kit and IL-10 Quantikine^®^ High sensitivity kit (both from R&D Systems). The range of the standard curve was 0.48–1500 pg/mL for IL-6 and 0.17–50 pg/mL for IL-10. Interassay CVs for the methods were 11.6 and 16.7% for IL-6 and IL-10, respectively. Samples were run in duplicates.

### Salivary cortisol

Salivary cortisol was collected with Salivette cotton swabs (Sarstedt, Nümbrecht, Germany), placed under the tongue for 2 min, on 3 consecutive days (not the day after Sunday or holiday, or the day before weekend). The morning sample was taken 30 min after awakening and the evening sample before bedtime. Participants were instructed not to eat, drink, or use tobacco for at least 60 min prior to saliva sampling. The Salivettes were immediately frozen at -20°C by the participants.

Levels of free cortisol in saliva were determined by a commercial radioimmunoassay assay, CORT-CT2 (Cisbio Bioanalyser, Codolet, France) at the accredited Clinical Chemical Laboratory at Karolinska University Hospital, Stockholm, Sweden. According to repeatedly performed quality assessments, the interassay CV was less than 10%.

### Statistical analyses

IBM SPSS Statistics 22 was used for statistical analyses, and for generation of graphs, Graph Pad Prism 5 was used. Chi-square test was used for nominal data. Kruskal-Wallis test was used to assess differences between all groups (SCAD patients, ACS patients and healthy controls), and Mann-Whitney U-test was used to test differences between 2 groups. Wilcoxon signed-ranks test was used for pair-wise comparisons and Friedman´s test was used to compare more than 2 observations repeated on the same subjects. Associations among variables were studied by means of Pearson´s correlation coefficient analysis of log transformed data. P < 0.05 was considered to be statistically significant. Values are presented as median and inter-quartile range.

## Results

### Characteristics of study participants

Characteristics of controls, SCAD patients and ACS patients are shown in Table 1. No major differences were seen regarding age, sex distribution, body mass index, smoking status, or salivary cortisol levels (the latter not available for ACS patients). Statins were used extensively in the SCAD population. In ACS patients, both leukocyte and monocyte numbers were higher compared with SCAD patients and controls while NK cell numbers were lower in both patient groups. Also, the levels of IL-6 were significantly higher in ACS patients. IL-10 in plasma did not differ across groups but levels were in general very low (and in 29% of the samples non-detectable).

**Table 1:** Characteristics of ACS patients, SCAD patients and controls.

	ACS (n = 10)	SCAD (n = 57)	Controls (n = 41)	*P*-value
Age, years	68 (61–73)	66 (61–71)	67 (66–72)	0.098
Female, n (%)	3 (30)	13 (23)	11 (27)	0.838
Current smokers, n (%)	1 (10)	6 (11)	1 (2.4)	0.304
Body mass index, kg/m^2^	28 (24–33)	27 (25–30)	26 (25–28)	0.253
Hypertension, n (%)	6 (60)***	29 (51)***	5 (13)	**<0.001**
Diabetes, n (%)	4 (40)**	12 (21)	0 (0)	**0.001**
Coronary angiography, 0/1/2/3-vessel disease, n ^a^	1/3/3/3	3/18/18/18	-	0.952
Statin treatment (> 2 months), n (%)	4 (40)*	56 (98) ***	4 (10)	**<0.001**
Total cholesterol, mmol/L	4.8 (4.1–5.7)	3.9 (3.3–4.3)***	4.6 (4.6–5.9)	**<0.001**
LDL cholesterol, mmol/L	3.2 (1.6–3.7)	2.1 (1.7–2.5)***	3.1 (2.5–3.9)	**<0.001**
HDL cholesterol, mmol/L	1.2 (1.0–1.4)**	1.1 (0.9–1.4)***	1.6 (1.4–1.8)	**<0.001**
Triglycerides, mmol/L	1.4 (0.9–2.1)	1.2 (0.9–1.8)	1.1 (0.8–1.7)	0.417
Morning mean saliva cortisol, nmol/L ^b^	-	37 (31–43)	40 (31–48)	0.268
Evening mean saliva cortisol, nmol/L ^c^	-	6.3 (4.9–7.9)	5.9 (5.0–8.0)	0.603
IL-6, pg/mL	14 (14)**	2.7 (2.2)	3.1 (2.9)	**0.003**
IL-10, pg/mL	0.36 (0.25–0.55)	0.31 (0.24–0.40)	0.31 (0.26–0.43)	0.818
Leukocytes, x 10^3^ cells/μL	8.4 (6.7–13)**	6.4 (5.1–7.5)	6.2 (5.3–7.3)	**0.031**
Monocytes, cells/μL	621 (448–779)*	479 (372–593)	473 (376–570)	**0.024**
CD4^+^ T cells, cells/μL	878 (687–1653)	861 (649–1105)	973 (757–1144)	0.296
CD8^+^ T cells/μL	513 (302–792)	444 (346–552)	475 (309–603)	0.851
NK cells, cells/μL	213 (156–323)***	196 (151–252)***	366 (242–486)	**<0.001**

Values are given as median (interquartile range), if nothing else is stated.

^**a**^ = Number of vessels with significant stenosis (≥70%)

^**b**^ = Taken 30 min after awakening, mean values from 3 consecutive days

^**c**^ = Taken before bedtime, mean values from 3 consecutive days

Kruskal-Wallis p-values are shown in the right column. For comparison between two groups Mann-Whitney U-test was used:

* *p* < 0.05 compared with controls,

** *p* < 0.01 compared with controls,

*** *p* < 0.001 compared with controls

### Expression of AnxA1 mRNA and protein in monocytes and lymphocyte populations

**Fig 1:**
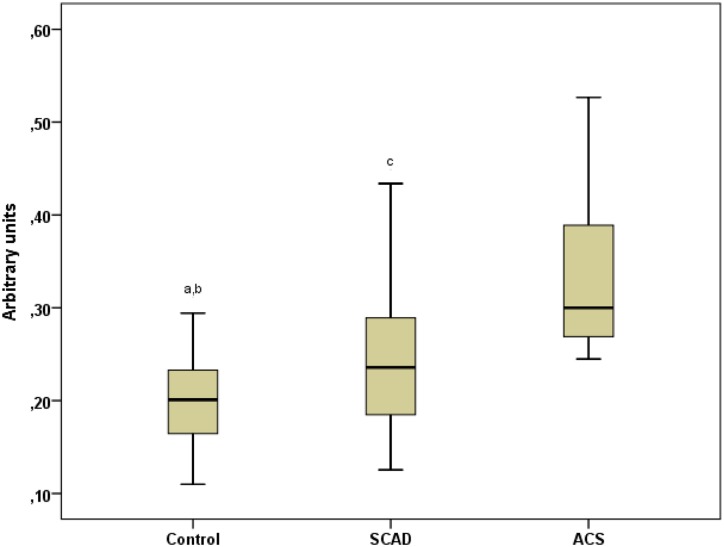
Expression of AnxA1 mRNA in isolated PBMCs from ACS patients, SCAD patients and controls. AnxA1 mRNA expression was analyzed in PBMCs collected immediately after density gradient isolation from 31 healthy controls, 49 SCAD patients and 10 ACS patients. The AnxA1 mRNA expression is presented as arbitrary units and was calculated using the ΔΔCT method. Each sample was run in duplicates. Box-and-whisker plots show median and interquartile range. Kruskal-Wallis test was used to assess differences between all groups, p < 0.001, and Mann-Whitney U-test was used for comparisons between 2 groups; ^a^ p < 0.05 vs SCAD, ^b^ p < 0.001 vs ACS, ^c^ p < 0.01 vs ACS.

**Fig 2:**
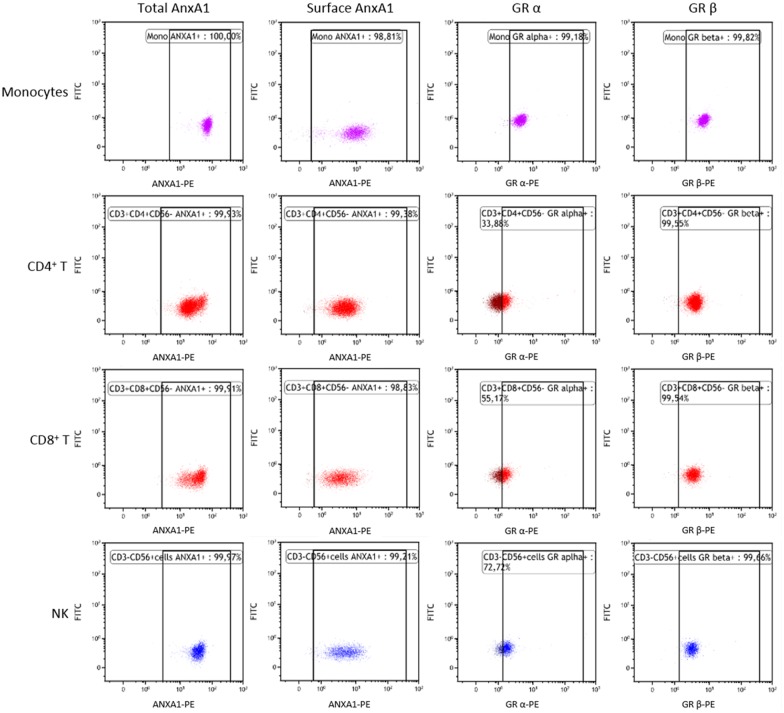
Representative examples of dot plots. The figure shows expression of total AnxA1, surface-bound AnxA1, GR-alpha and GR-beta, respectively (from left to right) for monocytes, CD4^+^ T cells, CD8^+^ T cells, and NK cells (from top to bottom).

**Fig 3:**
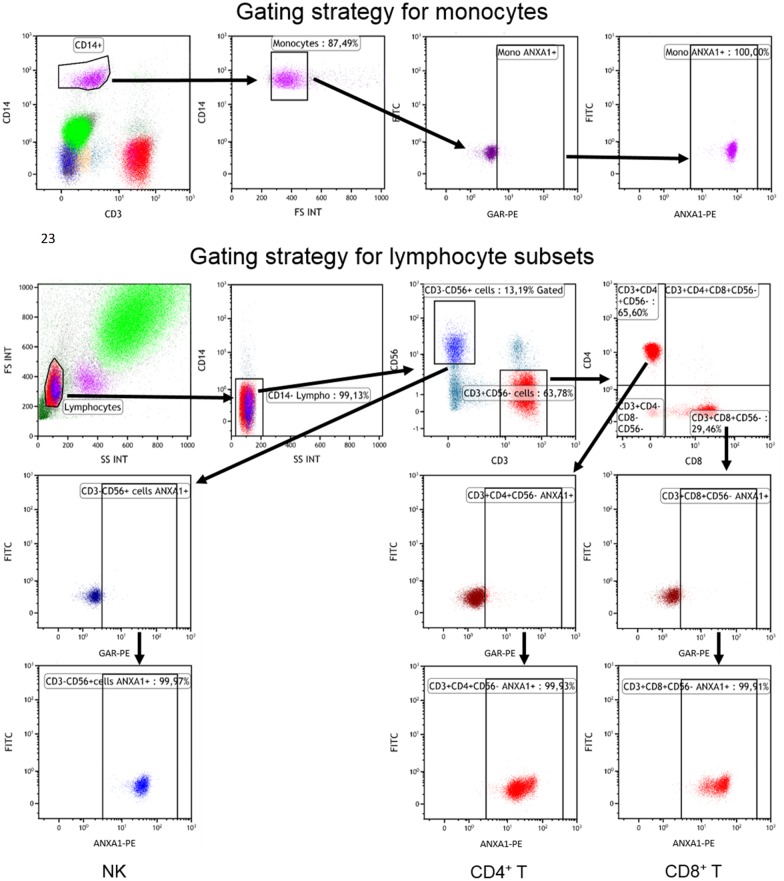
Representative examples of dot plots. The figure shows the gating strategy for total AnxA1. Monocytes were defined as CD3^-^CD14^+^ and further checked for size based on forward scatter (FS) before being plotted for expression of secondary goat anti-rabbit F(ab’)_2_(GAR)-PE. Lymphocytes were gated based on FS and side scatter (SS). Due to decrease in cell size and granularity as a result of intracellular staining (most prominently seen when stained for GR expression), lymphocytes were also defined as CD14^-^ to avoid inclusion of monocytes in lymphocyte gate. From CD14^-^ lymphocytes, NK cells were defined as CD3^-^CD56^+^, and T cells as CD3^+^CD56^-^. T cells were subsequently plotted for CD4 and CD8. For each subpopulation (monocytes, NK cells, CD4^+^ T cells and CD8^+^ T cells), the gate for AnxA1/GR-alpha/GR-beta expression was set visually as close to the main negative population as possible in the sample containing only the secondary GAR-PE antibody. The same gate was then kept for the sample containing the primary antibody.

The levels of AnxA1 mRNA in PBMCs were significantly higher in SCAD patients than in controls (Fig 1). However, in ACS patients, the levels were markedly higher compared with SCAD patients and controls. The flow cytometric assessments showed that AnxA1 protein was ubiquitously expressed in monocytes, T cells and NK cells but also that protein expression of total as well as surface AnxA1 was at least 2-fold higher in monocytes than in lymphocytes (Table 2 and Fig 2). A comparison between SCAD patients and controls did not reveal any significant difference in total protein levels of AnxA1 per cell type. The surface expression of AnxA1 was significantly higher in ACS patients than in SCAD patients and controls, as regards both monocytes, CD4^+^ T cells and NK cells (Table 2). Also, the total expression of AnxA1 in monocytes was higher in ACS patient than in SCAD patients. Similar gating strategies were used for total and surface-bound AnxA1 in monocytes and lymphocyte subsets. Representative dot plots are shown in Fig 3.

**Table 2:** Flow cytometric determination of the expression of total AnxA1 (surface + cytosolic), surface AnxA1 and Glucocorticoid Receptor (GR)-alpha and GR-beta in monocytes, CD4^+^ T cells, CD8^+^ T cells and NK cells in ACS patients, SCAD patients and controls.

	Leukocyte subset	ACS (n = 10)	SCAD (n = 54)	Controls (n = 37)	*P*-value
**Total AnxA1**	Monocytes	85 (70–93) #	62 (53–83)	67 (52–86)	0.118
	CD4^+^ T cells	26 (21–35)	20 (17–25)	20 (17–26)	0.106
	CD8^+^ T cells	39 (22–46)	27 (21–37)	28 (21–38)	0.327
	NK cells	37 (29–44)	28 (24–35)	31 (22–40)	0.240
**Surface AnxA1**	Monocytes	9.6 (8.4–9.9) ***#	7.9 (6.6–9.3)	7.4 (6.3–8.4)	**0.028**
	CD4^+^ T cells	4.3 (3.7–4.8) *#	3.7 (3.0–4.4)	3.6 (2.9–4.3)	0.052
	CD8^+^ T cells	3.1 (2.9–3.5) ***#	2.7 (2.2–3.3)	2.4 (2.1–2.8)	**0.000**
	NK cells	4.4 (3.6–5.0) ***#	3.5 (2.5–4.4)	3.1 (2.4–3.4)	**0.000**
**GR-alpha**	Monocytes	2.4 (2.2–2.7)*** ^§^	4.4 (3.6–6.3)	4.1 (3.3–5.8)	**0.000**
	CD4^+^ T cells	0.7 (0.6–0.7)*** ^§^	1.0 (0.9–1.3)	0.9 (0.8–1.1)	**0.000**
	CD8^+^ T cells	0.7 (0.6–0.7)*** ^§^	1.1 (0.9–1.5)	1.0 (0.8–1.3)	**0.000**
	NK cells	0.8 (0.7–0.9)*** ^§^	1.2 (1.0–1.9)	1.2 (1.0–1.5)	**0.000**
**GR-beta**	Monocytes	5.0 (4.7–5.9)	4.9 (3.8–6.5)	4.9 (3.9–6.1)	0.744
	CD4^+^ T cells	3.1 (2.6–3.6)	2.7 (2.0–3.6)	2.8 (2.1–3.3)	0.432
	CD8^+^ T cells	2.7 (2.3–3.4)	2.4 (1.8–3.1)	2.4 (1.9–3.0)	0.491
	NK cells	2.3 (2.2–3.0)	2.3 (1.7–3.1)	2.4 (1.9–2.6)	0.877

Mean fluorescence intensity values are given as median (interquartile range).

Kruskal-Wallis p-values are shown in the right column. For comparison between two groups Mann-Whitney U-test was used:

* *p* < 0.05 compared with controls,

** *p* < 0.01 compared with controls,

*** *p* < 0.001 compared with controls,

^#^ *p* < 0.05 compared with post-ACS,

^§^ *p* < 0.001 compared with SCAD.

### Expression of GR-alpha and GR-beta in monocytes and lymphocyte populations

GR-alpha and GR-beta were ubiquitously expressed in monocytes, T cells and NK cells. As shown in Table 2 and Fig 1A, both GRs were more abundantly expressed in monocytes than in lymphocyte subsets. The expression of GR-alpha did not differ between SCAD patients and controls whereas it was significantly lower in ACS patients. The expression of GR-beta did not differ across groups. The gating strategies GR-alpha and GR-beta were similar as described above for AnxA1 in monocytes and lymphocyte subsets (Fig 2B).

### Cytokine secretion ex vivo

The cytokine profile of PBMCs was assessed ex vivo in SCAD patients and controls. As shown in Table 3, the secretion of IL-1beta, IL-6, TNF and IL-10 was negligible or very low in the absence of LPS and increased markedly in both groups upon stimulation (p < 0.001 compared with non-stimulated PBMCs). PBMCs from SCAD patients secreted significantly more IL-6 than PBMCs from controls whereas there were no significant differences in LPS-stimulated secretion of IL-1beta, TNF or IL-10.

**Table 3:** Concentrations of IL-6, IL-1beta, TNF and IL-10 (pg/mL) in supernatants of PBMCs from 55 SCAD patients and 31 controls.

		SCAD (n = 55)	Controls (n = 31)	*P*-value
IL-6	Baseline	19 (6.1–33)	11 (0.0–34)	0.253
	LPS	19723 (11350–30604)	11369 (8549–17823)	**0.007**
	LPS + D^-8^	3518 (2378–5002)	4033 (3363–8029)	0.355
	LPS + D^-7^	4197 (3057–6491)	2682 (2203–5637)	0.163
IL-1beta	Baseline	0.0 (0.0–3.8)	0.0 (0.0–0.0)	0.630
	LPS	2905 (1501–4383)	2018 (1305–4143)	0.233
	LPS + D^-8^	1148 (752–1615)	878 (661–1643)	0.324
	LPS + D^-7^	692 (468–1130)	547 (363–1075)	0.291
TNF	Baseline	6.1 (0.0–9.6)	0.0 (0.0–6.3)	0.084
	LPS	1592 (1094–2031)	1542 (786–2149)	0.538
	LPS + D^-8^	956 (636–1361)	886 (611–1307)	0.453
	LPS + D^-7^	568 (405–734)	441 (323–628)	0.101
IL-10	Baseline	3.2 (0.0–4.7)	3.0 (0.0–4.1)	0.794
	LPS	840 (659–1150)	780 (568–892)	0.130
	LPS + D^-8^	451 (309–652)	386 (309–492)	0.220
	LPS + D^-7^	363 (258–469)	281 (208–368)	**0.022**

Cells were either unstimulated or stimulated with 100 ng/mL lipopolysaccharide (LPS) for 19 h. Dexamethasone was added to LPS-stimulated cells at two different concentrations, 10^−7^ M (D^-7^) or 10^−8^ M (D^-8^). Mann-Whitney U-test was used for comparison between patients and controls.

### Effects of dexamethasone on cytokine secretion ex vivo

Dexamethasone was added to PBMCs in order to test the glucocorticoid sensitivity ex vivo. In both patients and controls, dexamethasone markedly reduced the LPS-induced cytokine secretion (Table 3). The suppressive effect of dexamethasone, i.e. glucocorticoid sensitivity, was defined as follows: difference in cytokine level between LPS and LPS+dexamethasone treatment divided by LPS treatment and finally multiplied by 100. The higher dose of dexamethasone (10^−7^ M) induced a slightly stronger inhibitory effect on IL-6 secretion in patients than in controls, i.e. LPS-induced IL-6 levels were suppressed by 76 (68–83) % and 73 (62–78) %, respectively, p = 0.044. On the other hand, the suppression of IL-10 levels was slightly weaker in patients, 58 (50–67) % *vs* 64 (59–68) %, p = 0.023. The suppressive effects of dexamethasone on IL-1beta and TNF did not differ between groups.

### Effects of dexamethasone on AnxA1 mRNA expression ex vivo

The effect of LPS with or without dexamethasone on AnxA1 mRNA in PBMCs was assessed ex vivo. Treatment with LPS alone or LPS + dexamethasone 10^−8^ M for 19 h did not affect the expression of AnxA1 mRNA, given as percentage change compared with medium only, -3.4 ((-16)-26) % and –6.4 ((-22)-12) %, respectively, both NS. On the other hand, treatment with LPS + dexamethasone 10^−7^ M resulted in a significant increase in AnxA1 mRNA compared with medium only, +24 (1.6–59) %, p < 0.001. Results were similar in PBMCs from SCAD patients or controls, therefore results are shown for the whole study sample (n = 97).

### Correlations

**Fig 4:**
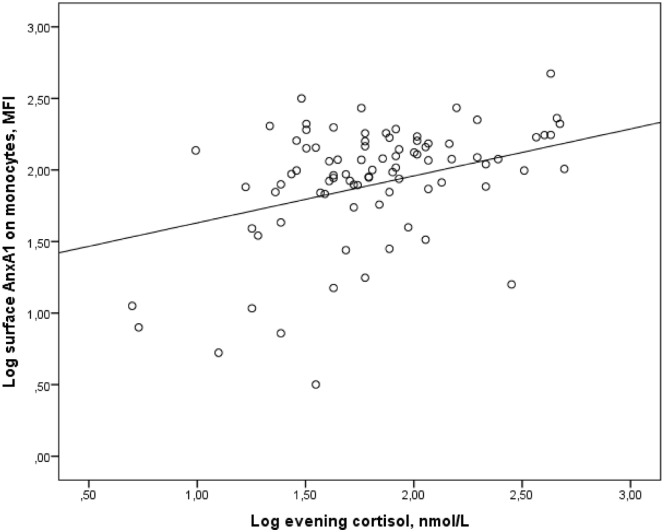
Correlation between surface AnxA1 expression on monocytes and evening cortisol levels. The figure shows the correlation between log transformed values of MFI values of surface AnxA1 expression on monocytes in peripheral blood and salivary evening cortisol, *r* = 0.298, p = 0.005.

**Fig 5:**
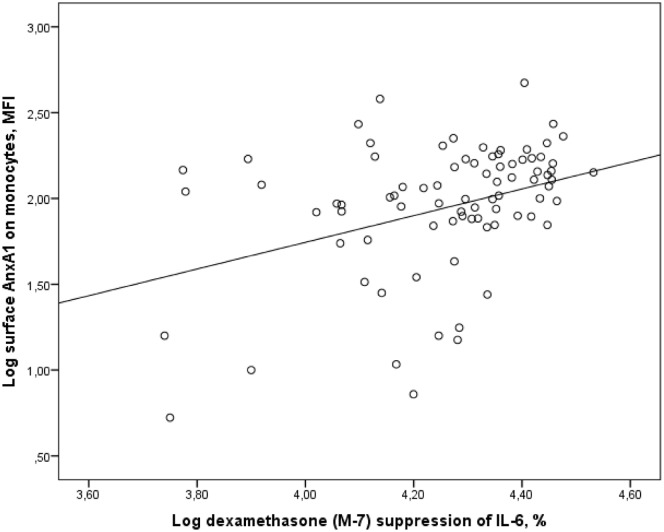
Correlation between surface AnxA1 expression on monocytes and glucocorticoid sensitivity. The figure shows the correlation between log transformed values of MFI values of surface and glucocorticoid sensitivity ex vivo, assessed by dexamethasone (M-7) suppression of LPS-stimulated IL-6 secretion from PBMCs (% of LPS-stimulated levels), *r* = 0.340, p = 0.002.

There were no significant correlations between factors such as age, sex and BMI and expression of AnxA1 or glucocorticoid sensitivity. The AnxA1 mRNA levels in PBMCs correlated with inflammatory status, assessed by IL-6 in plasma, *r* = 0.215, p = 0.034, and LPS-induced secretion of IL-6 and IL-1beta, *r* = 0.233, p = 0.027, and *r* = 0.212, p = 0.045, respectively. Neither AnxA1 mRNA, nor total AnxA1 protein levels showed correlations with cortisol levels or glucocorticoid sensitivity. On the other hand, the surface expression of AnxA1 protein in monocytes correlated with morning cortisol, *r* = 0.265, p = 0.013, and evening cortisol levels, *r* = 0.240, p = 0.024 (Fig 4). The surface expression of AnxA1 protein in monocytes also correlated with sensitivity to glucocorticoids ex vivo; dexamethasone-induced cytokine suppression of IL-6 by 10^−7^ M dexamethasone, *r* = 0.340, p = 0.002 (Fig 5). There were no correlations between GR-alpha or GR-beta expression and AnxA1 expression, inflammatory status or glucocorticoid sensitivity.

## Discussion

In the present study, we found that the PBMC expression of AnxA1 mRNA was significantly higher in SCAD patients than in healthy controls. This increase was however modest in relation to the marked increase that was observed in ACS, a condition characterized by acute inflammatory response. Moreover, ACS patients exhibited a significantly larger amount of AnxA1 protein on the surface of leukocytes, predominantly monocytes. The surface expression of AnxA1 is an indicator of enhanced activation of the AnxA1 pathway. In order to achieve its biological effects, the protein is translocated from the cytosol to the cell surface where it binds to a G protein-coupled receptor belonging to the formyl peptide receptor family [23]. Glucocorticoids are known to induce both de novo synthesis and translocation of AnxA1 to the cell surface [6, 9–11] and there is consistent evidence that AnxA1 is a key mediator of anti-inflammatory actions in monocytes/macrophages [4, 5]. Although the effects of AnxA1 has been studied mainly in vitro using exogenous AnxA1, a previous in vivo gene expression profiling study of human PBMCs verified that AnxA1 was a candidate marker of inflammatory modulation [24]. As expected, systemic inflammatory markers were markedly elevated in the ACS patients. The increased gene expression of AnxA1 together with the increased translocation of AnxA1 protein to the monocyte surface may thus represent an acute glucocorticoid action aiming to modulate the acute inflammatory response. This theory was further supported by the ex vivo findings that higher doses of dexamethasone (10^−7^ M) induced a significant increase in AnxA1 mRNA when added to LPS-stimulated PBMCs.

There were no differences in total or surface AnxA1 protein expression between SCAD patients and healthy controls. The SCAD patients were all clinically stable, not showing any signs of systemic inflammation as compared with controls. However, upon LPS stimulation their PBMCs released greater amounts of IL-6 suggesting the presence of a proinflammatory monocyte profile. This is consistent with previous reports showing that IL-6 secretion from LPS-stimulated PBMCs is enhanced in SCAD patients [25, 26]. One possibility is that the proinflammatory state of monocytes observed ex vivo is not strong enough to activate the AnxA1 pathway. A more speculative hypothesis is that the “normal” surface expression of AnxA1 on monocytes in SCAD patients reflects a diminished anti-inflammatory capacity in these patients. In a previous study, we showed that the neutrophil expression of AnxA1 protein was increased in SCAD patients (n = 30) compared with healthy controls (n = 30) and also, that this increase was associated with suppressed inflammatory properties of patients´ neutrophils [27].

It has been proposed that the low-grade chronic inflammation in patients with SCAD is associated with reduced sensitivity to glucocorticoids [20, 21]. However, we found no support for this hypothesis when we assessed the suppressive effects of dexamethasone on LPS-stimulated cytokine secretion. PBMCs from patients were at least as sensitive to dexamethasone as were PBMCs from controls. The glucocorticoid sensitivity did not correlate with AnxA1 mRNA nor with total AnxA1 protein levels. Instead, it correlated with the amount of AnxA1 located on the surface of circulating monocytes. The correlation between salivary cortisol levels and surface AnxA1 expression may further support the importance of surface AnxA1 as a marker of glucocorticoid sensitivity. In one earlier flow cytometry study, Mulla et al [28] showed a significant correlation between total AnxA1 protein expression in peripheral blood leukocytes and serum cortisol levels before and after a standard corticotrophin test. However, when they separated leukocytes according to size and granularity, the correlation between AnxA1 protein and glucocorticoid sensitivity was evident only for neutrophils, not for monocytes. In contrast to us, Mulla et al [28] did not perform measurements of surface AnxA1, which might explain the lack of correlations in monocytes in their study.

Alternative splicing in exon 9 generates two GR isoforms, GR-alpha and GR-beta. GR-alpha is the biologically active isoform while GR-beta is considered to be a negative inhibitor of GR. It has been proposed that upregulated expression of GR-beta is an indicator of glucocorticoid resistance, although data on this subject are not fully consistent [29]. As was previously shown by us, GR-beta expression in neutrophils did not differ between SCAD patients and controls [27]. In the present study, GR-beta expression in PBMCs did not differ between SCAD patients, ACS patients and controls, neither did it show any association with glucocorticoid sensitivity ex vivo. The reduced expression of GR-alpha that was observed in ACS patients may result from a negative feedback control of high cortisol levels. It is known that cortisol down-regulates the expression of GR-alpha [29].

Some limitations of this study warrant comment. The sample size of ACS patients was too small, neither were any cortisol measurements or glucocorticoid sensitivity tests performed in this group. Yet, we believe that ACS patients to some extent can fulfill the role of “reference” group for acute inflammatory response. In SCAD patients and controls, morning and evening cortisol were measured on 3 consecutive days. Still, the fluctuation of cortisol throughout the day makes it difficult to estimate an individual´s exposure to cortisol. In the glucocorticoid sensitivity ex vivo assays, we used LPS as a major stimulus for monocytes. Hence, we cannot draw any certain conclusions about the glucocorticoid sensitivity in T cells or NK cells.

To conclude, our findings indicate that flow cytometric determination of surface AnxA1 protein on peripheral monocytes can serve as a marker of glucocorticoid sensitivity, thereby reflecting anti-inflammatory capacity. However, there was no evidence for enhanced expression of AnxA1 protein in PBMCs from SCAD patients, despite them showing a proinflammatory monocyte profile ex vivo. Increased expression of AnxA1 in human atherosclerotic plaques has been associated with a stable plaque phenotype [17, 18]. One intriguing question for the future is whether the expression of AnxA1 protein in peripheral monocytes reflects the levels of AnxA1 protein in the atherosclerotic plaque and/or other characteristics of plaque stability.

## Supporting information

S1 File Data set. (SAV)
